# UPLC-MS/MS Profile Combined With RNA-Seq Reveals the Amino Acid Metabolism in *Zanthoxylum bungeanum* Leaves Under Drought Stress

**DOI:** 10.3389/fnut.2022.921742

**Published:** 2022-07-07

**Authors:** Haichao Hu, Xitong Fei, Beibei He, Xin Chen, Lei Ma, Peilin Han, Yingli Luo, Yonghong Liu, Anzhi Wei

**Affiliations:** ^1^College of Forestry, Northwest Agriculture and Forestry University, Xianyang, China; ^2^Research Centre for Engineering and Technology of Zanthoxylum State Forestry Administration, Xianyang, China; ^3^College of Horticulture, Northwest Agriculture and Forestry University, Xianyang, China

**Keywords:** *Zanthoxylum bungeanum* leaves, amino acid, metabolome, transcriptome, redundancy analysis

## Abstract

*Zanthoxylum bungeanum* leaves have a unique taste and incomparable nutritional value and hence are popular as a food item and traditional medicine in China. However, the studies on the metabolites in *Z. bungeanum* leaves are quite limited, especially for amino acids. Therefore, this study explored the amino acid component in *Z. bungeanum* leaves and also the accumulation mechanism under drought stress in two *Z. bungeanum* cultivars using the widely targeted metabolome combined with transcriptome analysis. A total of 56 amino acids and their derivatives were identified in *Z. bungeanum* leaves, including eight essential amino acids. The total amino acid content with most individual amino acids increased under progressive drought stress. More differentially accumulated amino acids (DAAs) and differentially expressed genes (DEGs) were found in FJ (*Z. bungeanum* cv. ‘Fengjiao’) than in HJ (*Z. bungeanum* cv. ‘Hanjiao’). The orthogonal projections to latent structures discriminant analysis identified nine and seven indicator DAAs in FJ and HJ leaves, respectively. The weighted gene co-expression network analysis (WGCNA) showed that the green module was significantly correlated with most indicator DAAs and revealed the important role of *FBA3, DELTA-OAT, PROC*, and 15 transcription factor genes in regulating the amino acid synthesis. Furthermore, the correlation analysis and redundancy analysis (RDA) identified four candidate synthesis genes (*ASNS*, *AK*, *ASPS*, and *PK*) in amino acid biosynthesis pathway. This study provided useful information for the development of *Z. bungeanum* leaves in food and nutrition industry and also laid the foundations for future molecular breeding.

## Introduction

*Zanthoxylum bungeanum* Maxim., a member of Rutaceae family, is an economically important plant. *Z. bungeanum* originated in East Asia and is now widely cultivated in China, Japan, South Korea, and Southeast Asian countries (1). As the main product, the peel of *Z. bungeanum* is an indispensable condiment in Chinese cuisine (1) and an important traditional Chinese herb with various therapeutic effects (2). The *Z. bungeanum* leaves are another important product of *Z. bungeanum*. In recent years, *Z. bungeanum* leaves have been shown to have a great potential as a food item because of their unique tingling taste, favorable aromatic flavor, and abundant nutrients. For example, the sprouts or young leaves of *Z. bungeanum* are usually eaten with other vegetables in a salad (3). They are also processed as canned pickles by the food industry for sale. The extract or volatile oil of *Z. bungeanum* leaves is used as a condiment and edible additive in foods (4). At the same time, the *Z. bungeanum* leaves can be made into green tea through the roasting process. Moreover, the extract of *Z. bungeanum* leaves can also be used as a food preservative (5). Therefore, *Z. bungeanum* leaves have broad prospects in the food industry.

Amino acids are the basic units in protein synthesis (6) and are also important raw materials for other secondary metabolites, such as terpenoids, flavonoids, and alkaloids (7, 8). Currently, 22 amino acids for synthesizing proteins have been discovered, including 20 major amino acids and two amino acids encoded by termination codons. The amino acids that the human body needs but cannot synthesize by itself or whose synthesis rate cannot meet the needs of human metabolism are called essential amino acids (9). The United Nations Food and Agriculture Organization stipulates that the percentages of eight kinds of essential amino acids are as follows: leucine 17.2%, isoleucine 12.9%, valine 14.1%, lysine 12.5%, threonine 10%, methionine 10.7%, phenylalanine 19.5%, and tryptophan 3.1%. In addition, histidine is an essential amino acid during infancy because the body cannot synthesize this amino acid during this period (10). Appropriate intake of amino acids is of great significance for regulating human function and maintaining human health (6). Glutamate is an amino acid that can promote red blood cell production, improve brain cell nutrition, and activate the mind (11). Phenylalanine is the raw material for the production of epinephrine, thyroxine, and melanin. Phenylalanine and tyrosine together synthesize important neurotransmitters and hormones, which are involved in the body’s glucose metabolism and fat metabolism (12). Tryptophan can improve sleep quality by promoting the secretion of serotonin and melatonin, and regulate the body’s biological clock (13). Threonine mainly functions in the gut for synthesizing mucosal proteins and modulating the immune response of the gut (14). Lysine can promote protein absorption and utilization, improve nervous system function, and enhance immune system function (15).

Amino acids are important nutrients in most natural crops and are important evaluation indicators of crop quality. A large number of studies focused on the content, ratio, and synthesis mechanism of amino acid components in various plant products, such as tea leaves (16), *Ginkgo* leaves (17), walnut (18), and *Lycium barbarum* (19). However, the amino acid component and content in *Z. bungeanum* leaves were rarely mentioned. For plants themselves, the amino acid composition has important implications for regulating plant physiological processes, including plant growth, flowering, and fruiting (20), as well as responses to abiotic stresses (21). Besides, the content of amino acids is easily affected by the environment (21). With the deterioration of the global environment, researches on the quality and yield of economical plant products in harsh environments, including drought, low temperature, and salinization, have attracted increasing attentions (22). *Z. bungeanum* is mainly grown in arid and semi-arid regions. Hence, it is of great significance to study the effect of drought stress on the nutrient composition of leaves.

Recent years witnessed the successful application of multiple omics in investigating the mechanism of metabolite synthesis. For example, Zhang et al. (23) identified the sabinene synthase gene in citrus leaves and flowers using metabolic and transcript data. Liu et al. (24) explored the volatile compounds and revealed the inner metabolism for flavor in apple fruit through the integration of transcriptome and metabolite profiling. Li et al. (25) investigated metabolite accumulation and the molecular mechanisms of flavonoid biosynthesis in jujube leaves using metabolic and transcriptomic profiles. Moreover, Xia et al. (26) integrated metabolic profiling and transcriptome to unveil the pigment accumulation metabolism in *Lonicera japonica* flowers. Therefore, in this study, the integrative analysis of transcriptome and metabolome was used to investigate the amino acid content in the leaves of *Z. bungeanum* in response to drought with three specific objectives: (1) to uncover the amino acid components in *Z. bungeanum* leaves, (2) to explore the effects of drought on amino acid content in leaves of two *Z. bungeanum* cultivars with different tolerance, and (3) to reveal the molecular mechanism of amino acid metabolism in *Z. bungeanum* leaves. The results of this study might provide an insight for the development of *Z. bungeanum* leaves in the food industry and also a pivotal clue for the molecular breeding of *Z. bungeanum*.

## Materials and Methods

### Plant Material and Sample

The mature seeds of two *Z. bungeanum* cultivars (FJ, *Z. bungeanum* cv. ‘Fengjiao’; HJ, *Z. bungeanum* cv. ‘Hanjiao’) were obtained from the Pickly Ash Experimental Station of Northwest A&F University in Fengxian, Shannxi Province, China (33°59′6.55″N, 106°39′29.38″E). After cleaning and air-drying, the *Z. bungeanum* seeds were sown in a soil mixture of perlite, vermiculite, and chernozem, and then placed in a research greenhouse of Northwest A&F University in Yangling, Shannxi Province, China. After germination, the healthy seedlings were transplanted into cultivar pots, with one seedling per pot. Then, these seedlings were cultivated for 3 months in the greenhouse with temperature at 25 ± 2°C and soil moisture at 85% ± 1%. After that, 36 *Z. bungeanum* healthy seedlings of the same size were selected in each cultivar and the water supply was stopped for 15 days. The top three fully expanded mature leaves were sampled with liquid nitrogen on days 0 (D1), 6 (D2), 9 (D3), and 15 (D4) and stored in a −80°C freezer. Three biological replications in each sample were performed, with three seedlings in each replication.

### Total Amino Acid Content

The total amino acid content was determined using assay kits (BC1570, Beijing Solarbio Science & Technology Co., Ltd., Beijing, China) following the manufacturer’s protocols. In this study, 0.1 g leaf samples were ground into powder with liquid nitrogen and used for the total amino acid determination. The absorbance for all samples was measured at 570 nm using a microplate reader (Infinite M200pro, Tecan, Switzerland).

### Amino Acid Profile in *Zanthoxylum bungeanum* Leaves

The 24 *Z. bungeanum* leaf samples were vacuum freeze-dried with a freeze drier (Scientz-100F, Zhejiang, China) and then ground into powders using a grinding mill (MM 400, Retsch, Germany). One-hundred milligrams of the powders were fully mixed with 1.2 mL of 70% aqueous methanol (*v/v*) with a Vortex-6 (Kylin-Bell, Jiangsu, China). After standing for 12 h at 4°C, the homogenate was centrifuged at 12,000 rpm at 4°C for 10 min (5424R, Eppendorf Co., Shanghai, China). The supernatant was collected and filtered through a 0.22-μm organic nylon needle filter (SCAA-104, ANPEL, Shanghai, China) into a brown sample bottle.

The quantitative and qualitative analyses of amino acids were performed by Wuhan MetWare Biotechnology Co., Ltd. (Wuhan, China) (27). Ultra-performance liquid chromatography (UPLC) analysis was carried out on the Shimadzu Nexera X2 instrument (Shimadzu, Japan) with an Agilent SB-C18 column (1.8 μm, 2.1 × 100 mm). The solvent A (ultrapure water with 0.1% formic acid) and solvent B (acetonitrile with 0.1% formic acid) were used as mobile phases for amino acid metabolites. The procedure was set as follows: 0 min, 5%; 0–9 min, increased to 95%; 9–10 min, 95%; 10–11.10 min, reduced to 5%; and 11.10–14 min, 5%. The tandem mass spectrometry (MS/MS) analysis was performed using an Applied Biosystems 4,500 QTRAP instrument (ABI, MA, United States) equipped with an ESI turbo ion-spray interface. The ESI source was operated as follows: ion source (turbo spray), source temperature (550°C), and ion-spray voltage (5500 V/−4500 V).

### RNA Extraction and Sequencing

Biomarker Technologies Co., Ltd. (Beijing, China) performed the RNA sequencing of 24 *Z. bungeanum* leaf samples. Total RNA from the *Z. bungeanum* leaves was extracted following the instruction on a Tiangen RNA Pure kit for plants (Tiangen, Beijing, China). The integrity and concentration of extracted RNA were determined using an Agilent 2100 Bioanalyzer (Agilent Technologies, Inc., CA, United States). The RNA with an OD260/280 value between 1.8 and 2.2 and an OD260/230 value of more than 2.0 was of high quality. The high-quality RNA was used to construct cDNA libraries employing an NEBNext Ultra RNA Library Prep Kit for Illumina (New England Biolabs, MA, United States). The Illumina HiSeq 2500 platform (Illumina, Inc., CA, United States) was used to sequence the resulting libraries. HISAT2 (28) was used to efficiently align RNA sequencing reads to reference *Z. bungeanum* genome. StringTie was used to assemble the aligned reads and calculate the fragments per kilobase of transcript per million fragments mapped (FPKM) (29). Differential expression analysis between sample groups was performed using DESeq2 (30), and genes with fold change (FC) ≥ 2 and false discovery rate (FDR) < 0.01 were defined as differentially expressed genes (DEGs). The functions of DEGs were annotated using GO (Gene Ontology), KO [Kyoto encyclopedia of genes and genomes (KEGG) Ortholog database), KOG/COG (Clusters of Orthologous Groups of proteins], Nr (NCBI non-redundant protein sequences), Nt (NCBI non-redundant nucleotide sequences), Pfam (Protein family), and Swiss-Prot (a manually annotated and reviewed protein sequence database). The transcriptome raw data has been uploaded to the NCBI SRA database with the accession number PRJNA784034.^1^

### Co-expression Network Construction

Weighted gene co-expression network analysis (WGCNA) was performed using the R package (version: 1.70-3) based on the DEGs with a similarity threshold 0.5 (31). The contents of important differentially accumulated amino acids (DAAs) under drought stress were input as the trait file. The modules with a high correlation to amino acids were identified. The co-expression network was constructed using the top 150 core genes in the green module and visualized using Cytoscape 3.9.1 (Java 11.0.6).

### Quantitative Real-Time Polymerase Chain Reaction

The specific quantitative primers for quantitative real-time polymerase chain reaction (qRT-PCR) were designed using Primer Premier 6.0 (PREMIER Biosoft, CA, United States) and are provided in Supplementary Table 1. A Tiangen RNA Pure kit for plants (Tiangen, Beijing, China) was used to extract the total RNA from the 24 *Z. bungeanum* leaf samples. We measured the concentration and integrity of total RNA using a NanoDrop 2000 spectrophotometer (Thermo Scientific, DE, United States). The total RNA with high quality was used to synthesize first-strand cDNA using a PrimeScript RT reagent kit with gDNA Eraser (Takara Biotechnology Inc., Dalian, China). qRT-PCR was performed in a 10 μL volume using SYBR Green Premix *Pro Taq* HS qPCR Kit (Accurate Biotechnology Co., Ltd., Hunan, China). The operational procedure in a CFX96 Real-Time System (Bio-Rad Laboratories, Inc., CA, United States) was set as follows: 1 cycle at 98°C for 30 s; 38 cycles at 95°C for 5 s, 56°C for 30 s, and 72°C for 30 s; and 4°C for the remaining. The relative transcription abundance was calculated by the 2^–Δ^ ^Δ^ *^CT^* method (32). *ZbUBA* and *ZbUBQ* were selected as internal reference genes to balance the gene expression (33).

### Statistical Analyses

The experimental data were processed using SPSS 23.0 (SPSS Inc., IL, United States), and significant differences among samples were analyzed using Duncan’s test in a one-way analysis of variance (*P* < 0.05). Correlation analysis and hierarchical cluster heatmap analysis (HCA) were performed using the online software “Wu Kong” platform.^2^ Principal component analysis (PCA), orthogonal projections to latent structures discriminant analysis (OPLS-DA), and redundancy analysis (RDA) were performed using the OmicShare tools online platform.^3^ The column chart was illustrated using Origin2021 (Originlab, MA, United States). The heatmaps of gene expression and amino acid abundance were drawn using TBtools (v. 1.098696, JAVA, China). For each sample, three replicates were included.

## Results

### Accumulation of Amino Acid Metabolites in *Zanthoxylum bungeanum* Leaves Under Drought Stress

**FIGURE 1:**
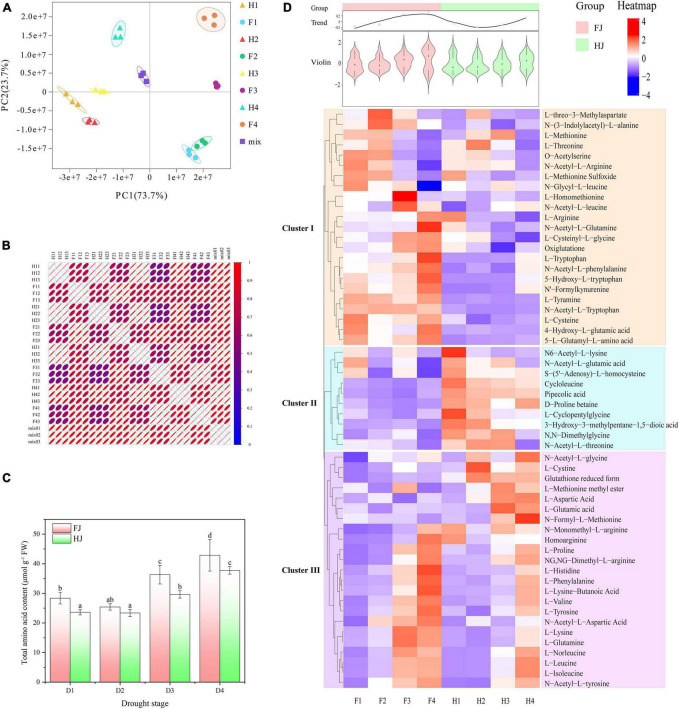
Amino acid profile analysis in ZB leaves under drought stress. **(A)** Principle component analysis (PCA) of 24 ZB leaf samples. **(B)** Intragroup correlation analysis of 24 ZB leaf samples. **(C)** Total amino acid content of ZB leaves under progressive drought stress. Red columns indicating the total amino acid content in ZB cultivar FJ; green columns indicating the total amino acid content in ZB cultivar HJ. D1, D2, D3, and D4 indicating the ZB leaves sampled 0, 6, 9, and 15 days after drought treatment. **(D)** Hierarchical clustering heatmap analysis (HCA) indicating the abundance of 56 individual amino acids in ZB leaves. Red and blue representing the high and low amino acid abundance, respectively. The above violin diagram and curve chart indicating the total variation in the 56 individual amino acids. F1, F2, F3, and F4 indicating the FJ samples in D1, D2, D3, and D4, respectively; H1, H2, H3, and H4 indicating the HJ samples in D1, D2, D3, and D4, respectively.

The qualitative and quantitative detection of amino acids were performed in 24 *Z. bungeanum* leaf samples to explore the amino acid metabolites in *Z. bungeanum* leaves. A total of 56 kinds of amino acids and derivatives, including eight essential amino acids, were identified (Supplementary Table 2). On the basis of the abundance of 56 amino acids, PCA was carried out on the 24 samples (including 3 replicates) (Figure 1A). The first two principle components explained 97.4% of the variation (PC1, 73.7%; PC2, 23.7%). FJ and HJ were separated along with PC1, and samples in different stages were separated by PC2. The results suggested that the abundance of amino acids was diverse in two *Z. bungeanum* cultivars, and drought stress had a large influence on amino acids in *Z. bungeanum* leaves. The intragroup correlation analysis showed a high correlation between biological replicates but a low correlation between cultivars, suggesting that the metabolite data were accurate and repeatable (Figure 1B). The total amino acid content increased with drought stress in both *Z. bungeanum* cultivars and was significantly higher in FJ than in HJ after D3 stage (Figure 1C). The HCA was performed to explore the variation in individual amino acid metabolite (Figure 1D). As a result, 56 amino acids were divided into three clusters: I, II, and III. Cluster I comprised 23 amino acid metabolites, including L-methionine, L-threonine, L-arginine, L-tryptophan, and L-cysteine. The contents of these amino acids were higher in FJ than in HJ. Likewise, 23 amino acids were contained in cluster III, including L-cystine, L-proline, L-histidine, L-valine, L-lysine, and L-leucine. Interestingly, the amino acids contents in both cultivars presented an increasing trend in cluster III (Supplementary Figure 1). In Cluster II, only 10 amino acids were present with higher content in HJ than in FJ. With regard to the total variation in 56 amino acids, the violin diagram on the basis of the content of 56 amino acids was illustrated. The total trend showed that the amino acids accumulated in both cultivars, especially after D3 stage, and the amino acids contents were higher in FJ than in HJ, which was in line with the total amino acid content.

### The Differentially Accumulated Amino Acids in Different Groups

**FIGURE 2:**
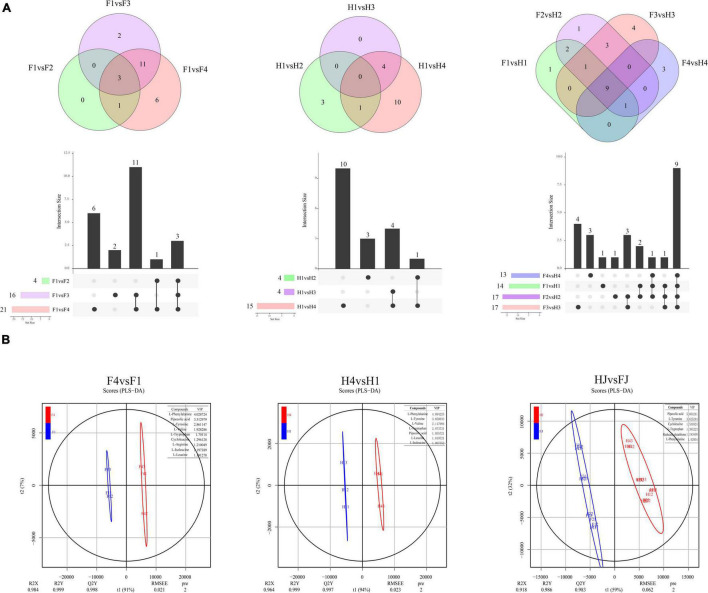
Differentially analysis of amino acid in ZB leaves. **(A)** Venn diagrams of differentially accumulated amino acids (DAAs). The below columns presented the numbers of each group. **(B)** Orthogonal projections to latent structures discriminant analysis (OPLS-DA) in F4vsF1, H4vsH1, and HJvsFJ. The table on the top right position in each diagram showed the indicator DAAs and the corresponding variable influence on projection (VIP) values.

The amino acids with FC > 2 or FC < 0.05 were defined as DAAs. Venn diagrams combined with column chart was used to depict differential metabolites in different comparison groups (Figure 2A). A total of 4, 16, and 21 DAAs were found in F1vsF2, F1vsF3, and F1vsF4, respectively. Interestingly, three DAAs (L-norleucine, L-histidine, and glutathione reduced form) were overlapped in three comparison groups. We speculated that these three amino acids might be particularly sensitive to drought, and thus their accumulation was disturbed in each stage of drought stress. A total of 4, 4, and 15 DAAs were found in H1vsH2, H1vsH3, and H1vsH4, respectively; no DAAs co-existed in the three comparisons. The fewer DAAs in HJ than in FJ implied that the amino acids in HJ were more stable under drought stress. A total of 14, 17, 13, and 17 DAAs were observed in F1vsH1, F2vsH2, F3vsH3, and F4vsH4, respectively. The result indicated differences in amino acid contents in *Z. bungeanum* leaves between the two cultivars.

We screened indicator DAAs with a variable influence on projection (VIP) > 1 using OPLS-DA, and these DAAs had a large contribution rate in the comparison group (Figure 2B). In F4vsF1, nine indicator DAAs were identified, namely, L-phenylalanine, pipecolic acid, L-tyrosine, L-valine, L-tryptophan, cycloleucine, L-arginine, L-isoleucine, and L-leucine, indicating that drought mainly affected the change in the amino acid content in FJ. In H4vsH1, seven important DAAs were L-phenylalanine, L-tyrosine, L-valine, L-tryptophan, pipecolic acid, L-leucine, and L-isoleucine, indicating that the seven amino acids were largely responsible for the variation in amino acids under drought stress in HJ. Notably, these seven amino acids were also included in important DAAs for FJ. In addition, six indicator DAAs (pipecolic acid, L-tyramine, cycloleucine, L-tryptophan, reduced glutathione, and L-phenylalanine) were found in HJvsFJ, indicating that these amino acids were the main reason for the difference in amino acids in *Z. bungeanum* leaves between two cultivars.

### Transcription Analysis in *Zanthoxylum bungeanum* Leaves Under Drought Stress

**FIGURE 3:**
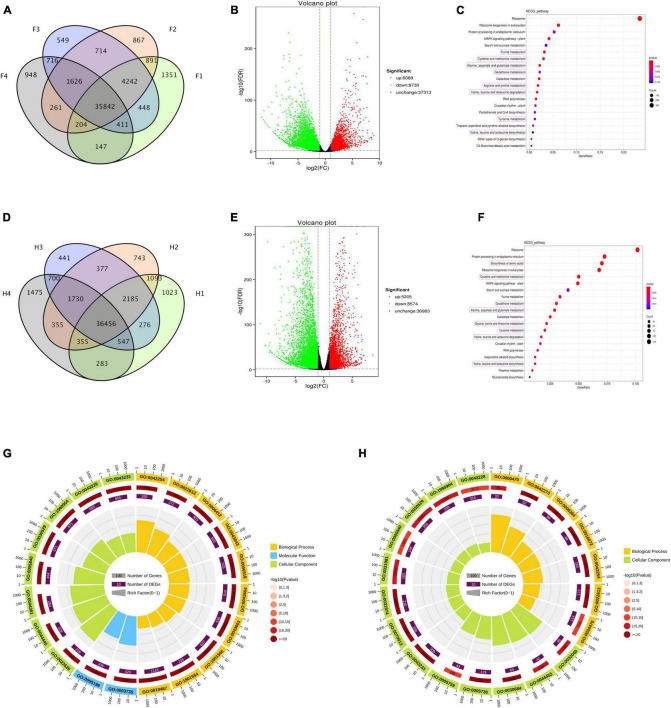
Analysis of differentially expressed genes (DEGs) in *Zanthoxylum bungeanum* leaves under drought stress. **(A,D)** Venn diagram of unigenes expressed at four drought stages in FJ and HJ, respectively. **(B,E)** Volcano plot of DEGs in FJ and HJ, respectively. Red dots indicating the upregulated genes, green dots indicating the downregulated genes, and black dots indicating the genes with no significant change. **(C,F)** Kyoto encyclopedia of genes and genomes (KEGG) enrichment bubble diagrams of DEGs in FJ and HJ, respectively. Bubble size indicating the number of DEGs enriched in this KEGG pathway; bubble color indicating the *P* values. **(G,H)** GO enrichment circle diagram of DEGs in FJ and HJ leaves, respectively. The outside lap represents the top 20 GO terms; the mid lap represents the numbers of all genes in GO terms and *P* values for gene enrichment for the specified GO term; and the inner lap represents the numbers of DEGs. The ladder column in the center represents the Rich factor of DEGs for each GO term.

The gene expression levels of two *Z. bungeanum* cultivars in four drought stages were analyzed using RNA-seq. A total of 85,942 genes were assembled and annotated in this transcriptome, and the Q30 base percentage in each sample was more than 92.96%. In FJ, 43,536, 44,647, 44,548, and 40,155 unigenes (FPKM > 1) were present in F1, F2, F3, and F4, respectively (Figure 3A), and 35,842 common genes were across the four periods. In HJ, 42,218, 43,294, 42,682, and 41,901 unigenes (FPKM > 1) were found in H1, H2, H3, and H4, respectively, and 36,456 genes existed commonly in the four periods (Figure 3D).

According to differential expression analysis between sample groups, the genes with FDR < 0.05 and a FC ≥ 2.0 were considered as DEGs. In FJ, 14,819 DEGs were identified under drought stress, with 6,089 genes upregulated and 8,730 genes downregulated (Figure 3B). In HJ, 13,779 DEGs were identified across the four stages, with 5,052 DEGs upregulated and 8,574 DEGs downregulated (Figure 3E). KEGG analysis and GO analysis were used to explore the possible functions of these DEGs. The KEGG bubble diagram showed the top 20 KEGG pathways enriched by these DEGs, eight of which were related to amino acid biosynthesis and mechanism in both cultivars (Figures 3C,F). Additionally, starch and sucrose metabolism and mitogen-activated protein kinase (MAPK) signaling pathways were also highly enriched. In FJ, the top 20 GO terms were divided into three categories, named as biological process (BP), molecular function (MF), and cellular component (CC), including translation (GO:0006412) and amide biosynthesis process (GO:0043604) in BP, structural constituent of ribosome (GO:0003735) and structural molecule activity (GO:0005198) in MF, and cytosolic ribosome (GO:0022626) in CC (Figure 3G). In HJ, only two GO classes were contained in the top 20 GO terms, including maturation of lsu-rRNA (GO:0000470) and ribosomal large subunit biogenesis (GO:0042273) in BP, and small-subunit processome (GO:0032040) and nucleolar part (GO:0044452) in CC (Figure 3H). In general, the DEGs under drought stress were related to the amino acid biosynthesis and metabolism, as well as plant signal transcription.

### Weighted Gene Co-expression Network Analysis of Differentially Expressed Genes

**FIGURE 4:**
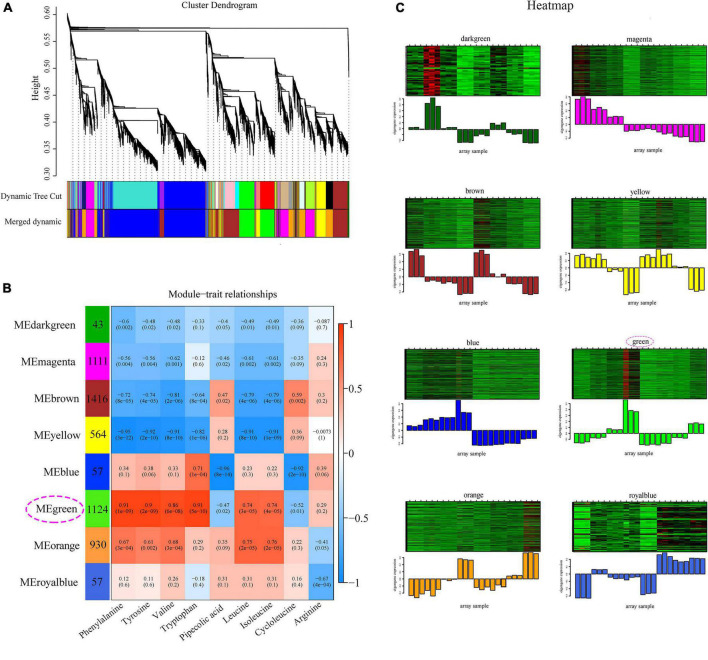
Weighted gene co-expression network analysis (WGCNA) of differentially expressed genes (DEGs) under drought stress. **(A)** Cluster dendrogram of all the DEGs. **(B)** Module–trait relationship heatmap of eight modules and nine indicator amino acids. **(C)** Gene expression heatmap of each module. The below column diagram represents the total variation trend of the genes in specific module.

Weighted gene co-expression network analysis was performed based on the DEGs combined with nine indicator DAAs in OPLS-DA under drought stress to investigate the molecular mechanism of amino acid biosynthesis in *Z. bungeanum* leaves under drought stress (Figure 4 and Supplementary Figure 1A). As a result, eight modules were produced according to the co-expression relationship (Figure 4B). More than 1,000 genes were observed in magenta, brown, and green modules; however, less than 100 genes existed in dark green, blue, and royal blue modules. Blue, green, and orange modules were positively related to most of these amino acids. Relatively higher correlation coefficients existed in green module, which significantly correlated to blue (*R* = 0.79, *P* = 0.002) and orange (*R* = 0.71, *P* = 0.04) modules (Supplementary Figure 1B). The overall gene expression pattern varied among the eight modules, which was visualized in a heatmap combined with a column chart (Figure 4C). Notably, the expression levels of most genes in the green module increased under drought stress and were higher in FJ than in HJ, which was in accordance to the varied trend in the contents of most amino acids. Taken together, the green module was selected as the most important module for further experimentation. The GO analysis showed that a large number of DEGs were enriched in metabolic process, response to stimulus and signaling in BP, cell and membrane in CC, and catalytic activity and transporter activity in MF (Supplementary Figure 2A). The KEGG analysis revealed that most DEGs in the green module were significantly enriched in plant hormone and signal transduction, MAPK signaling pathway, and biosynthesis and metabolism of multiple amino acids, such as cysteine and methionine metabolism, arginine and proline metabolism, and phenylalanine tryptophan tyrosine biosynthesis (Supplementary Figure 2B).

**FIGURE 5:**
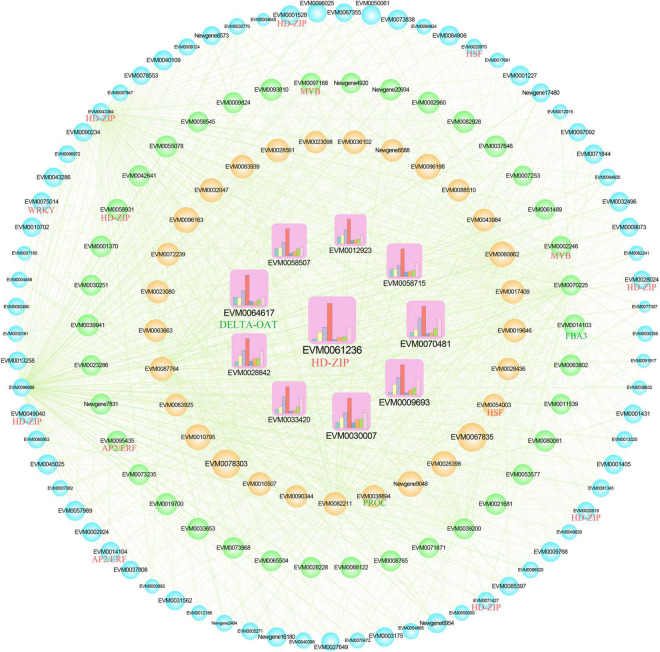
Co-expression network of the core 150 genes in the green module. The 10 hub genes are showed as squares with inner column chart of gene expression level. The other genes were illustrated as circles. The size of the shape represents the degree of connectivity. The green lines indicate significant correlations (*r* > 0.65). The transcription factor (TF) genes are labeled as red, and the structure genes annotated in the amino acid biosynthesis pathway are labeled as green. FBA3, fructose-bisphosphate aldolase 3; DELTA-OAT, ornithine aminotransferase; PROC, pyrroline-5-carboxylate reductase.

The gene co-expression network was constructed using the top 150 core genes in the green module (Figure 5). Among the 150 genes, three members were involved in amino acid biosynthesis pathway, named *fructose-bisphosphate aldolase 3* (*FBA3*), *ornithine aminotransfera* (*DELTA-OAT*), and *pyrroline-5-carboxylate reductase* (*PROC*). Transcriptional factors (TFs) play important roles in regulating the expression level of target genes. A total of 15 TF genes were found in the co-expression network, including two *AP2/ERF*s (*EVM0014104* and *EVM0095435*), eight *HD-ZIP*s (*EVM0001528*, *EVM0020518*, *EVM0028024*, *EVM0043364*, *EVM0049040*, *EVM0058931*, *EVM0061236*, and *EVM0071427*), two *HSF*s (*EVM0020970* and *EVM0054003*), two *MYB*s (*EVM0002246* and *EVM0097168*), and one *WRKY* (*EVM0075014*). Further, 10 hub genes were screened out according to the connectivity, including *DELTA-OAT* and *HD-ZIP*; their expression level increased with drought stress, which is shown by the column chart. Therefore, the 3 structure genes and 15 TF genes might play important roles in amino acid accumulation in *Z. bungeanum* leaves. Additionally, the expression patterns of these 18 genes were analyzed using qRT-PCR (Supplementary Figure 3). The relative expression levels of these 18 genes were consistent with FPKM values in RNA-seq, which validated the reliability of our transcriptome data.

### Gene Expression Level and Amino Acid Content in the Amino Acid Biosynthesis Pathway

**FIGURE 6:**
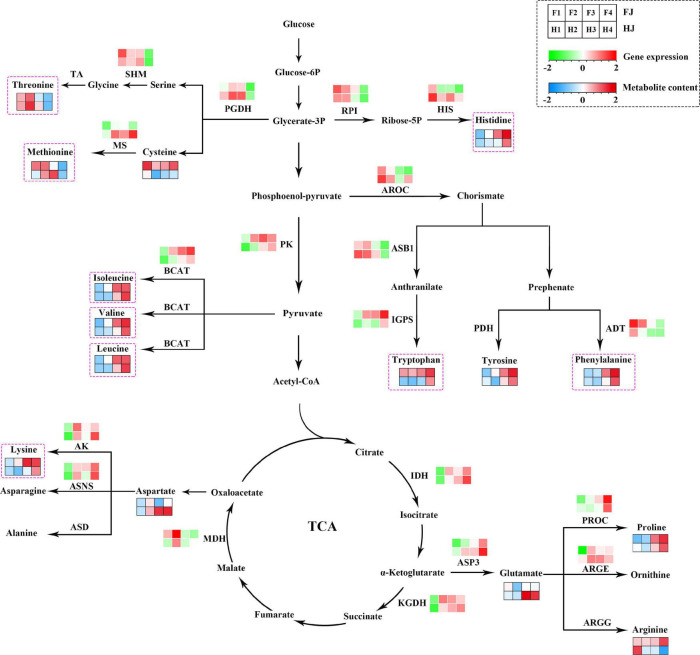
Amino acid biosynthesis pathway in *Zanthoxylum bungeanum* leaves. The green and red rectangles represent the heatmaps of gene expression; the blue and red rectangles represent the heatmaps of metabolite abundance. Pink dotted box marks the nine essential amino acids for human body. IDH, isocitrate dehydrogenase; PGDH, D-3-phosphoglycerate dehydrogenase; PROC, pyrroline-5-carboxylate reductase; MS, 5-methyltetrahydropteroyltriglutamate –homocysteine methyltransferase; SHM, serine hydroxymethyltransferase; BCAT, branched-chain-amino-acid aminotransferase; PK, pyruvate kinase 1; AK, aspartokinase; ARGE, acetylornithine deacetylase; IGPS, indole-3-glycerol phosphate synthase; ASB1, anthranilate synthase beta subunit 1; HIS, imidazoleglycerol-phosphate dehydratase; AROC, chorismate synthase; RPI, ribose-5-phosphate isomerase; ASNS, asparagine synthetase; ADT, arogenate dehydratase/prephenate dehydratase 6; ASP3, aspartate aminotransferase 3; TA, threonine aldolase; PDH, prephenate dehydrogenase; ASD, aspartate 4-decarboxylase; ARGG, argininosuccinate synthase.

A pathway diagram with gene expression and amino acids abundance was constructed in *Z. bungeanum* leaves according to the amino acid biosynthesis pathway reported in model plants (Figure 6). A total of 15 amino acids and 19 DEGs were mapped to the pathway. The contents of threonine, cysteine, and methionine decreased in both cultivars under drought stress. Consistently, the expression levels of *D-3-phosphoglycerate dehydrogenase* (*PGDH*) and *serine hydroxymethyltransferase* (*SHM*) also decreased. The histidine content was high under drought; however, a decreased expression level was presented in *imidazoleglycerol-phosphate dehydratase* (*HIS*) and *ribose-5-phosphate isomerase* (*RPI*). The contents of isoleucine, valine, and leucine increased with the increased expression levels of *pyruvate kinase 1* (*PK*) and *branched-chain-amino-acid aminotransferase* (*BCAT*). Differently, the tryptophan accumulated with the increased expression of *indole-3-glycerol phosphate synthase* (*IGPS*), and the contents of tyrosine and phenylalanine increased with the decreased expression of *arogenate dehydratase/prephenate dehydratase 6* (*ADT*). Tricarboxylic acid (TCA) cycle is an important respiration pathway whose intermediate products also participate in amino acid synthesis. For example, oxaloacetate was the product of aspartate and lysine; a-ketoglutarate was converted into glutamate by aspartate aminotransferase 3 (ASP3), and glutamate could be further converted into proline and arginine. In *Z. bungeanum* leaves, the lysine content increased with the higher *aspartokinase* (*AK*) gene expression level. The content of proline and glutamate increased with the elevated expression of *ASP3* and *PROC*. Generally speaking, most amino acids varied in accordance with the genes encoding synthesis enzymes despite several exceptions.

### Correlation Analysis and Redundancy Analysis Between Amino Acid Content and Expression of Synthesis-Related Genes

**FIGURE 7:**
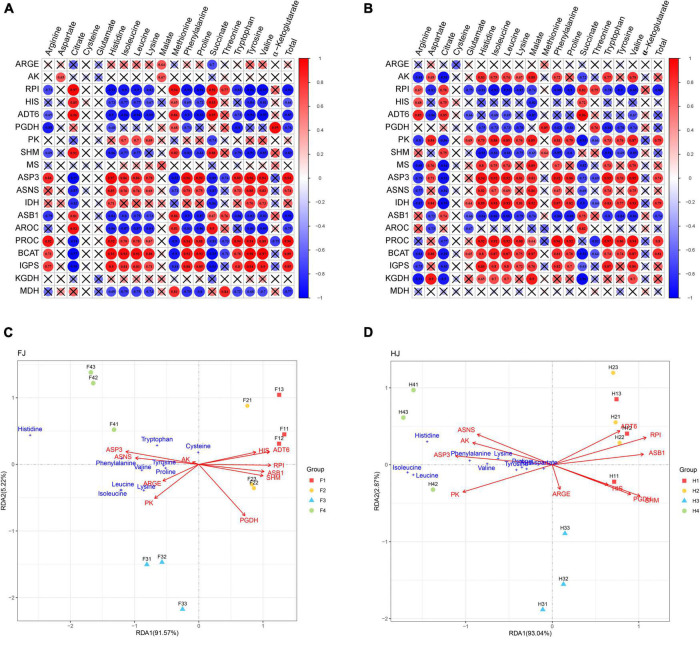
Correlation analysis and redundancy analysis (RDA) of amino acids and synthetic genes in amino acid biosynthesis pathway. **(A,B)** Intergroup analysis of amino acids and synthetic genes in FJ and HJ, respectively. **(C,D)** RDA of amino acids and synthetic genes in FJ and HJ, respectively.

The intergroup correlation between amino acid content and related genes in the biosynthesis pathway was analyzed in FJ and HJ to further explore the important genes involved in amino acid synthesis (Figures 7A,B). In FJ, the gene expression of *ASP3*, *ASNS*, *PROC*, *BCAT*, and *IGPS* positively correlated to the content of most amino acids, including arginine, histidine, isoleucine, leucine, lysine, phenylalanine, proline, tryptophan, tyrosine, and valine as well as the total amino acid content. Methionine content highly correlated with the expression of *RPI*, *ADT6*, *SHM*, *ASB1*, *AROC*, and *MDH* (*R* > 0.8, *P* < 0.05), while threonine content positively correlated with the expression of *RPI*, *ASB1*, and *MDH*. In HJ, the gene expression levels of *AK*, *PK*, *MS*, *ASP3*, *ASNS*, *IDH*, *PROC*, *BCAT*, and *IGPS* were positively associated with the contents of most amino acids, including histidine, leucine, lysine, tryptophan, and valine. The arginine content positively correlated with the expression of *RPI*, *HIS*, and *ADT6*. The methionine content positively correlated with the expression of *PGDH* and *SHM*. Besides, the total amino acid content highly correlated with the expression of *PK*, *ASP3*, *IDH*, *PROC*, and *BCAT*. These genes with positive correlation might be important in the biosynthesis of amino acids. Notably, TCA might play important roles in amino acid biosynthesis in HJ rather than FJ because of the positive correlation of the expression of *MS* and *IDH* with the contents of most amino acids in the biosynthesis pathway.

RDA was widely used to reveal the important factors in metabolite synthesis. RDA was performed using the contents of 15 amino acids and the expression levels of 19 synthesis-related genes in the aforementioned pathway. The expression levels of the genes related to amino acid synthesis explained 96.79 and 95.91% of the contribution rate in FJ and HJ, respectively, indicating that the RDA results were reliable and repeatable. In both FJ and HJ, a remarkable separation was found among samples in D1, D3, and D4, which was not significant between D1 and D2. The results indicated that drought stress had a significant influence on amino acids after mid drought stage. In FJ, the expression levels of *ASP3* and *ASNS* mostly correlated with the contents of tryptophan, histidine, phenylalanine, tyrosine, valine, and proline. The expression levels of *ARGE* and *PK* were significantly associated with the contents of isoleucine, leucine, and lysine. Besides, *AK* might play an important role in cysteine synthesis due to the smallest angle. However, the expression level of the other six genes did not significantly correlate with the contents of the aforementioned amino acids. In HJ, the expression of *ASNS*, *AK*, *ASPS*, and *PK* positively correlated with the 10 amino acids. In general, *ASNS*, *AK*, *ASPS*, and *PK* played important roles in amino acid biosynthesis in *Z. bungeanum* leaves.

## Discussion

The young leaves and buds of *Z. bungeanum* are a popular food, with unique taste and abundant nutrition. The mature *Z. bungeanum* leaves can be served as a food condiment and a Chinese medicinal herb. So far, the understanding of the metabolic components in *Z. bungeanum* leaves is quite limited. This study revealed the amino acid profile in *Z. bungeanum* leaves using a wide-targeted metabolome and investigated the amino acid synthesis mechanism in *Z. bungeanum* leaves under drought stress by the conjoint analysis of the transcriptome and metabolome. The results increased our understanding of amino acid components in *Z. bungeanum* leaves, amino acid accumulation pattern under drought stress, and the corresponding mechanism, as well as the key genes regulating amino acid synthesis, thereby providing reference for plant-based food industry and laying the foundation for further molecular breeding.

Amino acids are the basic units of protein synthesis, which is of extraordinary significance to living organisms. Totally, 20 basic amino acids are involved in protein synthesis and eight of these are classified as essential amino acids according to the demand of human body, including leucine, isoleucine, lysine, methionine, tryptophan, valine, threonine, and phenylalanine. These essential amino acids need to be obtained from the daily diet. Therefore, understanding the amino acid composition in food is of great significance for a reasonable diet. The content and composition of amino acids in plant-based foods have been extensively studied, such as *Torreya grandis* (34), green tea (16), *Ginkgo* leaves (17), and date palm leaves (35). In this study, 56 types of amino acids and their derivatives, including eight essential amino acids and histidine, were identified in *Z. bungeanum* leaves using UPLC-MS/MS (Figure 1 and Supplementary Table 2). The result suggested that the *Z. bungeanum* leaves were rich in amino acids. The ingestion of *Z. bungeanum* leaves could help supplement the amino acid content in the human body.

Unlike animals, plants are stationary and cannot move to avoid unfavorable environments. In the long-term evolution process, higher plants have evolved different mechanisms to reduce the damage caused by unfavorable environment, and many of these mechanisms are related to amino acid metabolism (21). A general accumulation of free amino acids is usually observed in different plants exposed to drought stress; however, the reason for accumulation may be different for individual amino acids (36, 37). Consistently, the total free amino acid content increased especially in the mid and late drought stage in *Z. bungeanum* leaves (Figure 1C). The accumulation of proline and γ-aminobutyric acid (GABA) not only regulated the osmotic pressure but also served as reactive oxygen species scavengers to reduce oxidative toxicity (21, 38). Proline synthesis was significantly stimulated under drought stress (39). Additionally, glutathione (GSH), which functioned as an antioxidant, played numerous roles in plant cells, including stress tolerance, regulation of cellular redox balance, modulation of the expression of stress-related genes, and plant growth and senescence (40). The glutathione–ascorbate cycle is considered one of the most pivotal metabolic pathways for the detoxification of H_2_O_2_ (41). The overexpression of GSH biosynthesis genes enhanced the drought tolerance in transgenic plants (42). Besides, the exogenous application of GSH was successful in increasing the plant tolerance to drought stress (43). In *Z. bungeanum* leaves, the contents of both proline and reduced glutathione were upregulated under drought conditions (Figure 1D), suggesting that the upregulated amino acid content contributed to the drought tolerance in *Z. bungeanum*. In addition, amino acids are precursors of some signaling molecules that help regulate other substances to regulate drought responses. For example, arginine is the precursor substance of polyamines, which is important in drought response and widely investigated in many crops (44). Lysine can be converted into immune signaling molecule *N*-hydroxy pipecoline (45). In FJ leaves, the contents of these two amino acids were upregulated under drought stress. Furthermore, our transcriptome analysis found that the DEGs related to amino acid synthesis were largely enriched in plant hormone and signal transduction (Supplementary Figure 2).

Differential analysis of amino acid metabolites showed more DAMs in FJ than in HJ at three drought stages, which was in agreement with DEGs (Figures 2, 3). This result indicated that HJ, the tolerant *Z. bungeanum* cultivar, had a more stable homeostatic system under drought conditions, thus reducing the disturbance from adverse environments. OPLS-DA was used to screen out the DAMs with a significant contribution to the differential group, and these were considered as indicator DAMs (46). The OPLS-DA identified nine indicator DAAs (L-phenylalanine, pipecolic acid, L-tyrosine, L-valine, L-tryptophan, cycloleucine, L-arginine, L-isoleucine, and L-leucine) in FJ and seven indicator DAAs (L-phenylalanine, L-tyrosine, L-valine, L-tryptophan, pipecolic acid, L-leucine, and L-isoleucine) in HJ (Figure 2B). We considered these DAAs to be the drought biomarkers in *Z. bungeanum* leaves. In addition, the amount of DAAs in the two cultivars was not significantly influenced by the drought stage, implying that the differences in amino acids existed among different *Z. bungeanum* cultivar leaves. An in-depth amino acids analysis in the leaves of different *Z. bungeanum* cultivars might be of great significance for *Z. bungeanum* reasonable utilization in the food and nutrition industry in the future.

Integrating metabolome and transcriptome is an effective tool to study metabolic mechanisms (47). Based on similar trends in the contents of indicator DAAs (input as the trait file) and the transcriptional metabolism levels of DEGs, a WGCNA was performed to explore the mechanism by which drought regulated amino acid accumulation. The green module significantly positively correlated with most indicator amino acids (Figure 4B). The functional annotation showed that the DEGs in this module were related to amino acid synthesis and metabolism (Supplementary Figure 2), indicating that WGCNA was meaningful for the amino acid changes in this study. In the green module, 150 core genes were selected to construct the co-expression network (Figure 5). Among these, three genes were annotated as amino acid–related genes, namely, *FBA3, DELTA-OAT*, and *PROC*. The results of both RNA-seq and qRT-PCR showed the increased trend of *FBA3, DELTA-OAT*, and *PROC*, which was consistent with the contents of phenylalanine, tyrosine, valine, tryptophan, leucine, and isoleucine as well as total amino acid content, indicating that these three genes were important for amino acid accumulation. TFs were proteins that could modulate the rate of gene transcription by binding to the 5′-upstream region of target genes (48). They played an important role in metabolite synthesis by regulating gene transcription, protein synthesis, and subsequent altered cellular function (34, 49). For example, proline accumulation was regulated by TFs associated with phosphate starvation (50). In this study, 15 TF genes were identified in the co-expression network, including two *AP2/ERF*s, eight *HD-ZIP*s, two *HSF*s, two *MYB*s, and one *WRKY*, suggesting the possible regulatory role of these TFs in amino acid biosynthesis.

The amino acid biosynthesis pathway is complex and a cross-talk of C metabolism and N metabolism (17). Most of the basic amino acids are biosynthesized using the intermediates of the Embden–Meyerhof pathway and the TCA cycle as the carbon chain backbone, apart from aromatic amino acids and histidine (51). Aromatic amino acids are derived from erythrose-4-phosphate, an intermediate of pentose phosphate pathway, and histidine is biosynthesized using ATP and phosphoribosyl pyrophosphate (51). Based on the data in the transcriptome and metabolome, we constructed an amino acid biosynthesis pathway in *Z. bungeanum* leaves under drought conditions (Figure 6). Among the 15 amino acids, most amino acid contents were consistent with their synthetic gene expression, with some exceptions, such as *ADT* and phelylalanine, *HIS* and histinidine, and *MS* and methionine. We speculated that the degradation and transformation downstream of these metabolites might be enhanced.

A large scale of secondary metabolites with multiple healthcare functions was derived from the shikimic acid pathway of aromatic amino acids (phenylalanine, tyrosine, and tryptophan) (52). Furthermore, phenylalanine is an important intermediate in the flavonoid synthesis pathway. In this study, drought induced the accumulation of these three aromatic amino acids in *Z. bungeanum* leaves, which provided more sufficient raw materials for synthesizing secondary metabolites. This was in line with the results of our previous studies, which showed an increase in the flavonoid content in *Z. bungeanum* leaves under drought conditions (53). Long-chain amino acids (isoleucine, leucine, and valine), which are important supplements for athletes (54), accumulated in the *Z. bungeanum* leaves under drought stress. Therefore, we speculated that *Z. bungeanum* leaves had the potential to develop sports drinks; however, more in-depth validation needs to be performed. On the whole, drought leads to an upregulation of most amino acids, including seven essential amino acids. The correlation analysis and RDA were carried out on the amino acid content and gene expression level in the amino acid biosynthesis pathway to explore the pivotal synthesis-related genes in amino acid biosynthesis (Figure 7). As a result, four amino acid synthesis–related genes having a significant positive correlation with most of the amino acids were sheltered, named *ASP3*, *ASNS*, *PK*, and *AK*. In a recent study, the overexpression of four unigenes positively correlated with the contents of at least 10 amino acids contributing to amino acid biosynthesis in *Torreya grandis* (34). Thus, these four genes can be used as candidate genes for improving amino acid content in *Z. bungeanum* leaves in future studies.

## Conclusion

In this study, 56 amino acids and their derivatives were identified in the *Z. bungeanum* leaves using UPLC-MS/MS, including eight essential amino acids. Drought stress caused an increase in the total amino acid content and the contents of most amino acids, including seven essential amino acids, which indicated that the drought environment increased the amino acid nutrition level in *Z. bungeanum* leaves. WGCNA identified three amino acid synthesis–related genes, *FBA3, DELTA-OAT*, and *PROC*, and 15 TF genes, which might participate in the biosynthesis of amino acids in *Z. bungeanum* leaves under drought conditions. In addition, the correlation analysis and RDA analysis screened out four candidate genes (*ASNS*, *AK*, *ASPS*, and *PK*) in amino acid biosynthesis in the leaves of two *Z. bungeanum* cultivars. This study provided useful information for the development of food nutrition and also important clues for the cultivation of high-amino acid *Z. bungeanum* leaves.

## Data Availability Statement

The datasets presented in this study can be found in online repositories. The names of the repository/repositories and accession number(s) can be found in the article/Supplementary Material.

## Author Contributions

HH: methodology, software, data curation, and writing—original draft. XF: supervision and writing—review and editing. BH: validation and investigation. LM and XC: data curation. PH and YLu: investigation. YLi: resources and supervision. AW: funding acquisition and resources. All authors contributed to the article and approved the submitted version.

## Conflict of Interest

The authors declare that the research was conducted in the absence of any commercial or financial relationships that could be construed as a potential conflict of interest.

## Publisher’s Note

All claims expressed in this article are solely those of the authors and do not necessarily represent those of their affiliated organizations, or those of the publisher, the editors and the reviewers. Any product that may be evaluated in this article, or claim that may be made by its manufacturer, is not guaranteed or endorsed by the publisher.

## Footnotes

1
https://dataview.ncbi.nlm.nih.gov/object/PRJNA784034?reviewer=v79bcipc97tajk7ppuam33d88t

2
https://www.omicsolution.com/wkomics/main/

3
https://www.omicshare.com/tools
