# Supplementary material for: UPLC-MS/MS Profile Combined With RNA-Seq Reveals the Amino Acid Metabolism in Zanthoxylum bungeanum Leaves Under Drought Stress
Source: Front Nutr. 2022 Jul 7;9:921742. doi: 10.3389/fnut.2022.921742 (PMC9301252; doi:10.3389/fnut.2022.921742)
Supplement: Supplementary file 1 [file Presentation_1.pdf]

Supplementary Material

Supplementary Figures

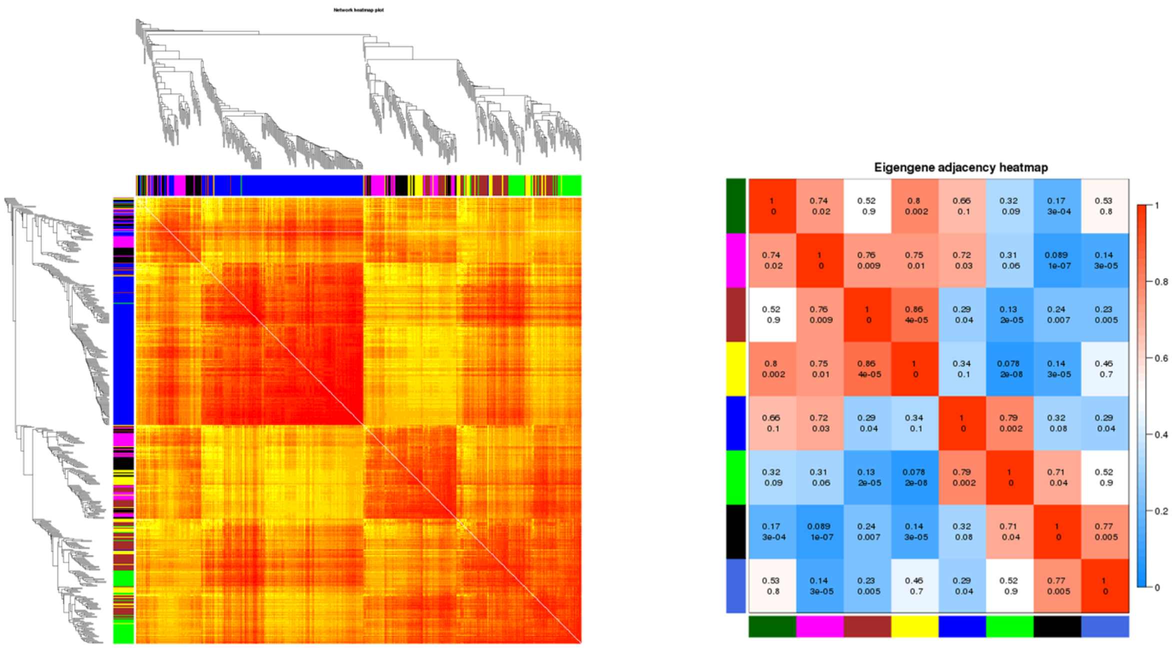

A **B**

Supplementary Figure 1. Weighted gene co-expression network heatmap of all DEGs (A) and correlation heatmap of eight modules (B).

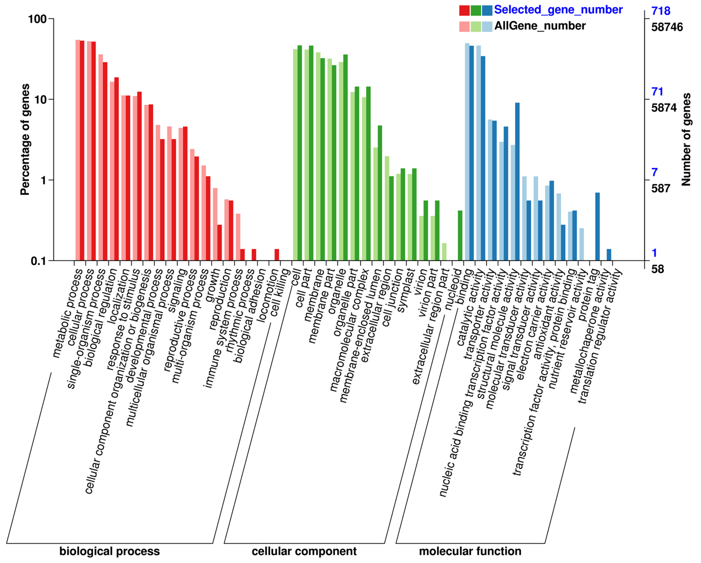

A

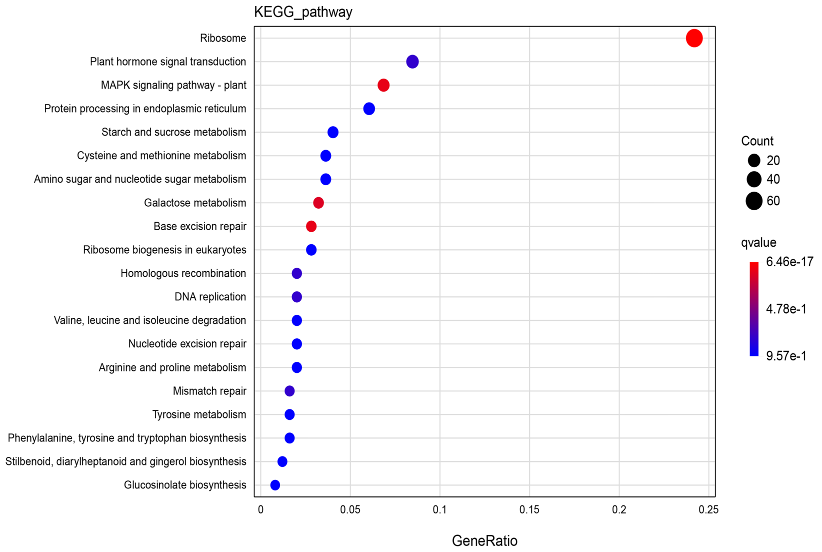

B

Supplementary Figure 2. Function enrichment analysis of DEGs in green module. A: GO enrichment analysis. B: KEGG enrichment analysis.

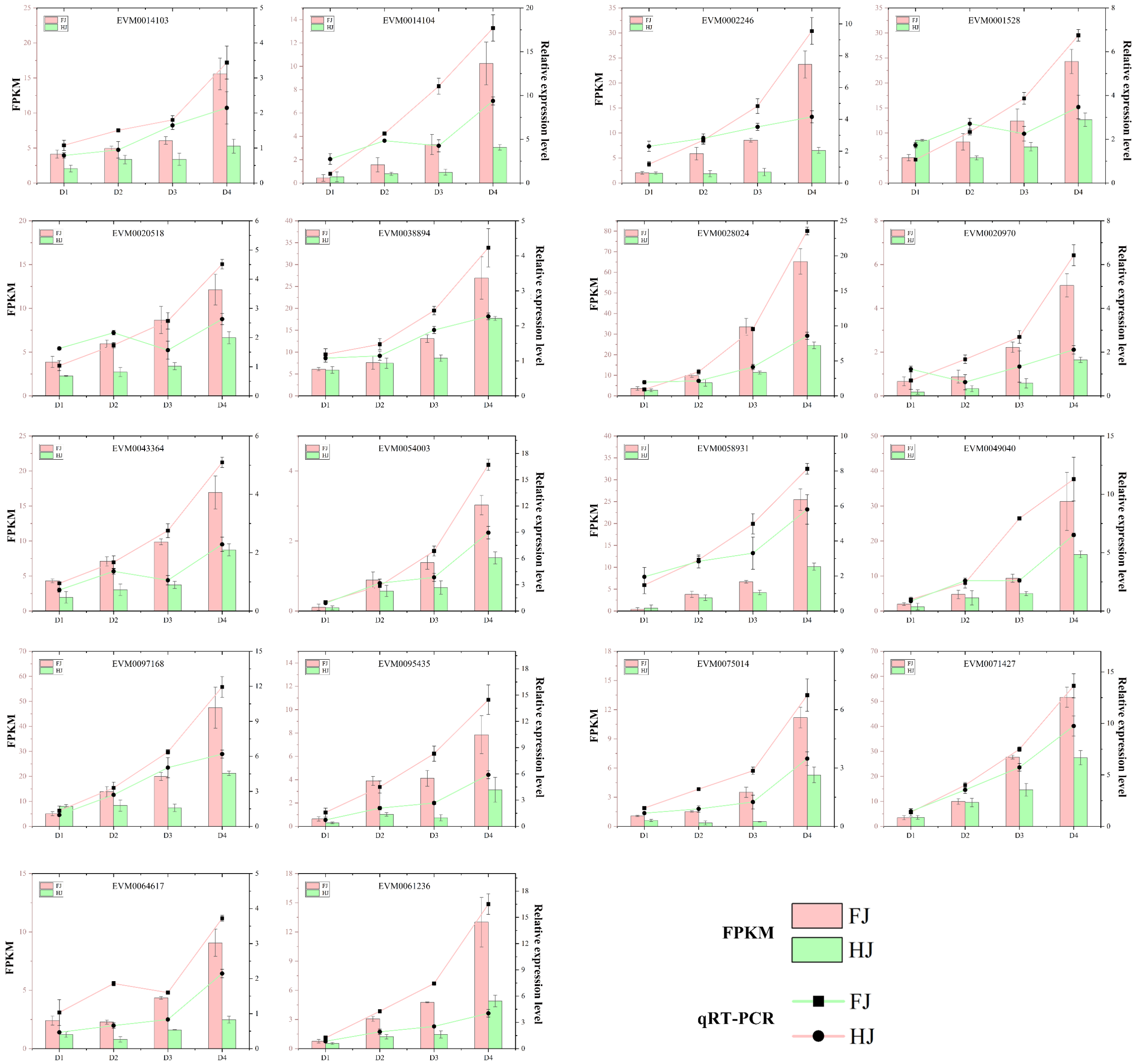

Supplementary Figure 3. qRT-PCR validation of 18 synthetic genes in amino acid biosynthesis pathway. The column chart shows the FPKM values in RNA-seq data. The line diagram represents the relative expression level in qRT-PCR.
